# Alpha-Fetoprotein+Alkaline Phosphatase (A-A) Score Can Predict the Prognosis of Patients with Ruptured Hepatocellular Carcinoma Underwent Hepatectomy

**DOI:** 10.1155/2022/9934189

**Published:** 2022-04-21

**Authors:** Feng Xia, Elijah Ndhlovu, Zhicheng Liu, Xiaoping Chen, Bixiang Zhang, Peng Zhu

**Affiliations:** Hepatic Surgery Center, Tongji Hospital of Tongji Medical College of Huazhong University of Science and Technology, Wuhan, Hubei, China

## Abstract

**Background:**

This research is aimed at establishing a scoring system alpha-fetoprotein+alkaline phosphatase (A-A score) based on preoperative serum alpha-fetoprotein (AFP) and alkaline phosphatase (ALP) levels and to investigate its clinical significance in patients with ruptured hepatocellular carcinoma (rHCC) after hepatectomy.

**Methods:**

175 ruptured hepatocellular carcinoma (HCC) patients treated with hepatectomy were included. Survival analysis was assessed by the Kaplan-Meier method. Prognostic factors were analyzed in a multivariate model. Preoperative serum AFP and ALP values are assigned a score of 1 if they exceed the threshold value and 0 if they are below the threshold value, A-A score is obtained by summing the scores of two variables (AFP, ALP), and the predictive values of AFP, ALP, and A-A score were compared by receiver operating characteristic curve (ROC) analysis, and subgroup analyses were performed to further evaluate the power of A-A scores.

**Results:**

Of the 175 patients, 67 (38.3%) had an A-A score of 0, 72 (41.1%) had an A-A score of 1, and 36 (20.6%) had an A-A score of 2. In multivariate analysis, the A-A score, the BCLC stage, and the extent of resection were independent predictors of OS in patients with rHCC. The 1-, 3-, and 5-year OS and RFS in patients with an A-A score of 1 were better than those with an A-A score of 0 and worse than those with an A-A score of 1 (all *p* < 0.05). Based on the results of ROC analysis, the A-A score is superior to AFP or ALP alone in predicting the prognosis of patients with ruptured HCC. In subgroup analysis, A-A score could accurately predict the prognosis of patients with or without microvascular invasion (MVI) and with different Child-Pugh grades or gender.

**Conclusions:**

The A-A score can effectively predict the prognosis of patients after hepatectomy of ruptured hepatocellular carcinoma. At the same time, it also has good evaluation ability in different subgroups.

## 1. Introduction

Hepatocellular carcinoma (HCC) is the 4th most common type of cancer in the world and the 2nd leading cause of death in all cancer types [1]. In some cases, patients with HCC will experience spontaneous rupture, and this is one of the most critical complications of HCC with a poor prognosis [2]. Many articles report an acute phase mortality rate of 25%–75% due to HCC rupture. However, with the early detection of more HCCs, the incidence of rupture is decreasing year by year. The incidence of spontaneous rupture of liver cancer varies widely geographically. There are differences between different countries, and the reason for this difference is different etiologies, such as hepatitis B and cirrhosis—of course, this situation occurs more in China. In our country, a large number of people are infected with hepatitis B, [3] which will eventually lead to HCC, and HCC patients in China account for nearly 50% of the world, which brings great pressure to local medical departments [4–6]. Complete hepatectomy is a potentially curative therapy for ruptured hepatocellular carcinoma (rHCC) [7].

Kirikoshi et al. [8] found that in all patients with rHCC, maximum tumor length of no more than 7 cm was the only independent factor determining long-term survival. Zhang et al. [9] found that maximum tumor length ≥ 10 cm and tumor noncapsule were independent prognostic factors for overall survival (OS) and recurrence-free survival (RFS).

Alpha-fetoprotein (AFP) is generally considered to be a relatively effective parameter for early diagnosis of HCC, and it is elevated in about 60-70% of all HCC patients. Many studies have shown that higher serum AFP levels are associated with aggravated malignancy of HCC cells, while lower AFP is associated with a lower degree of malignancy. Clinically, the measurement of serum AFP before surgery can effectively determine the prognosis after hepatectomy, while the levels of AFP can reflect the recovery of patients when they are reexamined postoperatively [10–13]. Meanwhile, Zhang et al. [13] found that AFP ≥ 1000 ng/mL was a risk factor for poorer survival outcomes and set the AFP cut-off value at 1000 ng/mL. However, some researchers have questioned this and suggested AFP may not be a good prognostic indicator because it is not elevated in 30% of HCC patients. Some researchers even suggest that AFP does not have a good ability to predict the postoperative prognosis [8, 14]. From these studies, we believe that when using AFP to predict the postoperative prognosis of ruptured HCC, it should be combined with other predictive markers.

Serum alkaline phosphatase (ALP) level is another biomarker related to HCC, and alkaline phosphatase is widely distributed in human tissues as an enzyme, which is metabolized by the liver and finally excreted in the bile [15].

In clinical work, the role of ALP may be underestimated relative to alanine aminotransferase (ALT) and aspartate aminotransferase (AST) because there are relatively few studies on ALP. A recent study [16] showed that ALP was a risk factor that affected progression-free survival. Therefore, this can be combined with AFP, to predict the prognosis after rHCC surgery. Albhaisi et al. [1] reported a nonlinear relationship between cancer mortality and serum ALT and AST levels, but higher ALP levels were associated with a higher risk of cancer death, and Xu et al. [17] used ALP to build a predictive model to predict the prognosis of HCC. These studies suggest that ALP can be used to further identify patients with poor prognoses if AFP is normal. To the best of our knowledge, no research has yet combined two markers to predict the prognosis after surgery for rHCC. Therefore, in this study, we aimed to establish a scoring system based on ALP and AFP to help predict the prognosis of ruptured HCC underwent hepatectomy.

## 2. Materials and Methods

### 2.1. Patients

Patients who underwent hepatectomy for spontaneous rupture of hepatocellular carcinoma at our hospital from January 2010 to March 2021 were extracted from the Department of Hepatic Surgery of our hospital. All liver procedures were performed by a surgical team with 15 years of experience, and our institution is one of the largest liver surgery centers in Asia, with various complex surgeries in the direction of the liver, which are routinely performed in our center. The variables obtained included patient gender, age, longest diameter and number of tumors, presence of portal hypertension, presence of microvascular invasion of the tumor, preoperative transcatheter arterial embolization (TAE), preoperative tyrosine kinase inhibitors (TKI), intraoperative regular excision, intraoperative extent of resection, BCLC stage of the tumor, pathological differentiation of the tumor, Child-Pugh grade of the tumor, preoperative AFP, preoperative ALP, presence of necrosis, and local excision of the tumor.

HCC diagnosis was confirmed by experienced pathologists in our hospital. The presence of MVI as well as other pathological features was extracted from the pathology report. We defined the inclusion criteria for patients as follows: (1) pathologically confirmed HCC, (2) single tumor, (3) Child grade A or B, and (4) tumor rupture determined by enhanced CT and abdominal MRI by experienced imaging physicians.

The exclusion criteria for patients were as follows: (1) patients with pathologically confirmed non-HCC, (2) positive surgical margins, (3) presence of lymph node metastasis and/or macrovascular invasion, (4) patients with recurrence and re-resection, and (5) incomplete follow-up information and clinical data. Microvascular invasion (MVI) is more common in small branches of the portal vein in the hepatic tissue; it can also be seen in the hepatic vein and occasionally in the bile duct, hepatic artery, and small branches of lymphatic vessels [18]. Satellite lesions are defined according to existing studies as lesions within 2 cm of the main body of the tumor [19]. Our research was authorized by the Ethics Committee of Wuhan Tongji Hospital, and all patients gave informed consent.

### 2.2. Definitions

**Figure 1:**
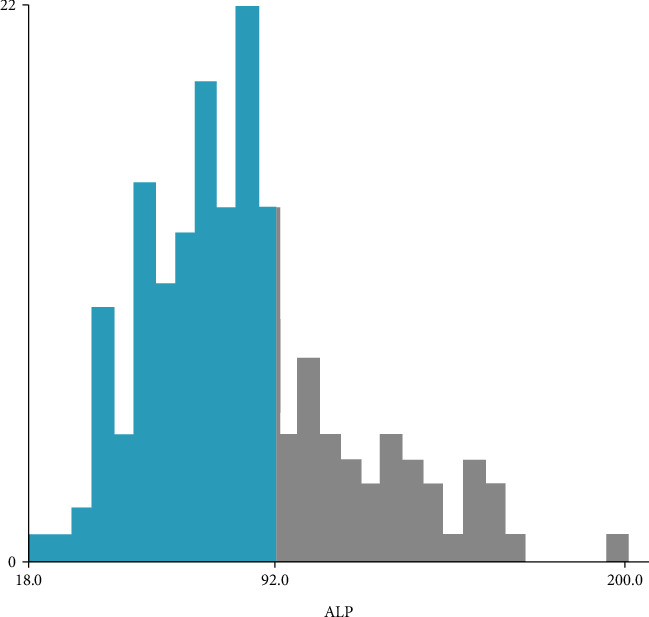
The cut-off value for the continuous variable ALP was determined by the X-tile software studied at Yale University (cut‐off = 92 U/L).

Surgery for rHCC is generally completed one week after admission, and serum AFP and ALP levels are measured at admission. As reported in the literature, the cut-off value for AFP is 1000 ng/mL. rHCC patients with AFP > 1000 ng/mL were given a score of 1. The optimal cut-off value was determined by X-tile software developed at Yale University [20], and serum ALP was dichotomized by overall survival. Patients with ALP > 92 U/L were given a score of 1 (Figure 1). The patients were divided into group A (A-A score 0), group B (A-A score 1), and group C (A-A score 2) based on the sum of the scores for AFP or ALP.

### 2.3. Follow-Up

According to the guidelines, we administer antiviral therapy in postoperative patients. All patients with rHCC were followed every quarter in the first year after surgery and every half year in the second year. During each follow-up, liver and kidney function, blood routine, blood biochemistry, imaging examination, including abdominal enhanced CT, were performed to determine whether there was tumor recurrence. If the possibility of recurrence was suspected, further abdominal MRI and even PET-CT were performed. For patients with recurrence, retreatment options include tumor reresection, transcatheter arterial chemoembolization (TACE), and radiofrequency ablation (RFA). Overall survival (OS) was defined as the time interval from the first day after surgery to the date of death or last follow-up. Recurrence-free survival (RFS) was defined as the time interval from the first day after surgery to the date of discovery of a neoplasm in the liver or other sites at the first postoperative reexamination or the date of the last follow-up.

### 2.4. Data Analysis and Expression

Measurement data were expressed as mean ± standard deviation (*x* ± *s*), and *t*-tests were performed for comparison. Enumeration data adoption rate (%) was expressed, and the comparison was tested by the Chi-square test. Kaplan-Meier method was used for OS and RFS, and Cox proportional hazards were used for multivariate analysis. ROC were used to compare the predictive discrimination and clinical utility of different variables.

SPSS 25.0 statistical software and R software (version 4.0.5, version 4.0.5, R Foundation for Statistical Computing, Vienna, Austria) was used for data processing. A value of *p* < 0.05 was defined as statistically significant.

## 3. Results

### 3.1. Basic Characteristics

**Figure 2:**
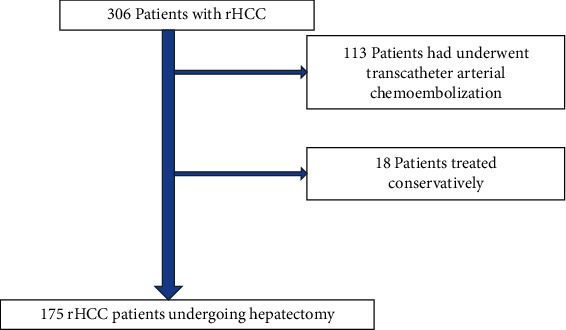
Flow chart about patients' selection.

175 patients with ruptured HCC were included in our study (Figure 2). Demographic and basic characteristics were shown in (Table 1). There were 153 males (87.4%) and 22 females (12.6%), with a mean age of 46 (38–54) years. MVI and local tumor necrosis were observed in 22 (12.6%) and 89 (50.9%) patients, respectively. Portal hypertension was observed in 43 (24.6%) cases. For the Edmondson-Steiner pathological grade, twenty patients (11.4%) had grade I tumors, 82 (46.9%) had grade II, 40 (22.9%) had grade III, and 33 (18.9%) had grade IV. The median tumor diameter for the entire cohort was 7.4 (5.0–10.2). 82 (46.9%) patients had AFP ≤ 1000 ng/mL, and 93 (53.1%) had AFP > 1000 ng/mL. 51 (29.1%) patients had ALP > 92 U/L. According to the results of combined AFP and ALP, the rHCC patients were divided into groups A, B, and C (A represents a score of 0, *n* = 67 (38.3%); B represents a score of 1, *n* = 72 (41.1%); and C represents a score of 2, *n* = 36 (20.6%)).

Regarding the presence of MVI, there were differences between the three groups. It can be seen from the proportions that more cases of MVI were present in the groups with higher A-A scores, indicating that the occurrence of MVI may be related to AFP or ALP. For BCLC stage, there were still differences among the three groups. When the A-A score was higher, the percentage of stages C and D was also higher. It could also be seen that a higher A-A score was associated with a worse prognosis; similarly, in the Edmondson pathological stage, when the A-A score was higher, the was a higher possibility of poor tumor differentiation.

### 3.2. A-A Score Has a Significant Relationship with the Postoperative Prognosis of Patients

The univariate screening for overall survival resulted in nine potential factors. In multivariate analysis, the extent of tumor resection (HR: 4.219, 95% CI: 1.889–9.421, *p* < 0.001), BCLC stage (HR: 1.359, 95% CI: 1.055–1.749, *p* = 0.017), and A-A score (HR: 1.871, 95% CI: 1.432–2.443, *p* < 0.001) were independent predictors of the OS (Table 2). Univariate regression of RFS screened out 10 potential factors and finally included three variables as shown in Table 3: tumor length (HR: 1.057, CI (95%): 1.007-1.110, *p* < 0.026), portal hypertension (HR: 1.497, 95% CI: 1.013-2.212, *p* = 0.043), and A-A score (HR: 1.789, 95% CI: 1.353-2.366, *p* < 0.001).

**Figure 3:**
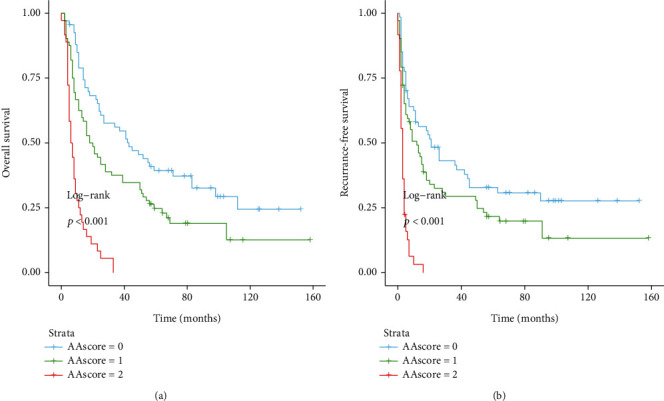
Survival analysis based on the A-A score. The 1-, 3-, and 5-year OS (a) and RFS (b) among patients with an A-A score of 1 were better than that of patients with an A-A score of 2 and worse than those of patients with an A-A score of 0 (all *p* < 0.0001). RFS: recurrence-free survival; OS: overall survival.

The 1-, 3-, and 5 year OS and RFS rates in group A were 73.6%, 55.2%, and 35.8% and 53.7%, 37.3%, and 23.9%, respectively. The 1-, 3-, and 5 year OS and RFS rates for group B were 62.5, 37.5, and 19.4%; and 47.2, 26.4, and 16.7%, respectively. The 1-, 3- and 5 year OS and RFS rates for group C were 25.0%, 0%, and 0%; and 2.8%, 0%, and 0%, respectively (Figure 3). In summary, the results of data analysis showed that patients with an A-A score of 1 had a better prognosis than those with an A-A score of 2 (RFS: *p* < 0.001; OS: *p* = 0.001), but worse than the group with a score of 0 (RFS: *p* < 0.001; OS: *p* = 0.01).

### 3.3. Comparison of A-A Score, AFP, and ALP Predictive Ability

**Figure 4:**
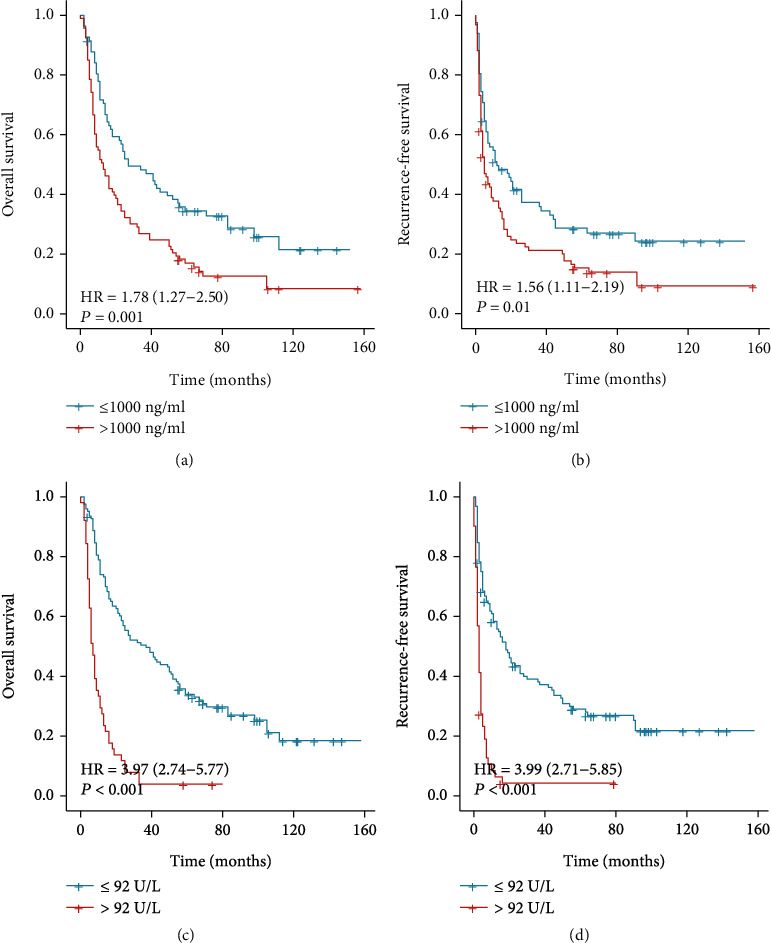
Survival analysis based on serum AFP or ALP. The 1-, 3-, and 5-year OS (a) and RFS (b) among patients with AFP ≤ 1000 ng/mL were better than that of patients with AFP > 1000 ng/mL (all *p* < 0.05). The 1-, 3-, and 5-year OS (c) and RFS (d) among patients with ALP ≤ 92 U/L were better than that of patients with ALP > 92 U/L (all *p* < 0.05). AFP: alpha-fetoprotein; ALP: alkaline phosphatase.

From the K-M survival curves, we found that the different preoperative AFP levels made a statistically significant difference in overall survival and recurrence-free survival between the two groups of rHCC patients (>1000 vs. ≤1000 ng/mL) (Figures 4(a) and 4(b)). The 1-, 3-, and 5 year OS rates were 70.7%, 47.6%, and 30.5%, respectively, in patients with preoperative AFP ≤ 1000 ng/mL and 51.6%, 26.9%, and 14.0%, respectively, in patients with AFP > 1000 ng/mL (*p* < 0.001). The 1-, 3-, and 5 year RFS rates were 47.6%, 22.0%, and 20.7%, respectively, in patients with AFP ≤ 1000 ng/mL and 34.4%, 19.4%, and 11.8%, respectively, in patients with AFP > 1000 ng/mL (*p* < 0.001). When divided according to preoperative serum ALP levels, the prognosis of patients with higher serum ALP (ALP > 92 U/L) levels was poorer (Figures 4(c) and 4(d)). The 1-, 3-, and 5-year OS rates were 73.4%, 50.0%, and 29.8%, respectively, in patients with ALP ≤ 92 U/L and 29.4%, 3.9%, and 2.0%, respectively, in patients with ALP > 92 U/L (*p* < 0.01). The 1-, 3-, and 5-year RFS of patients ≤92 U/L were 54.0%, 34.7%, and 21.8%, respectively, and 7.8%, 2.0%, and 0.0% in patients with ALP > 43 U/L (*p* < 0.01).

**Figure 5:**
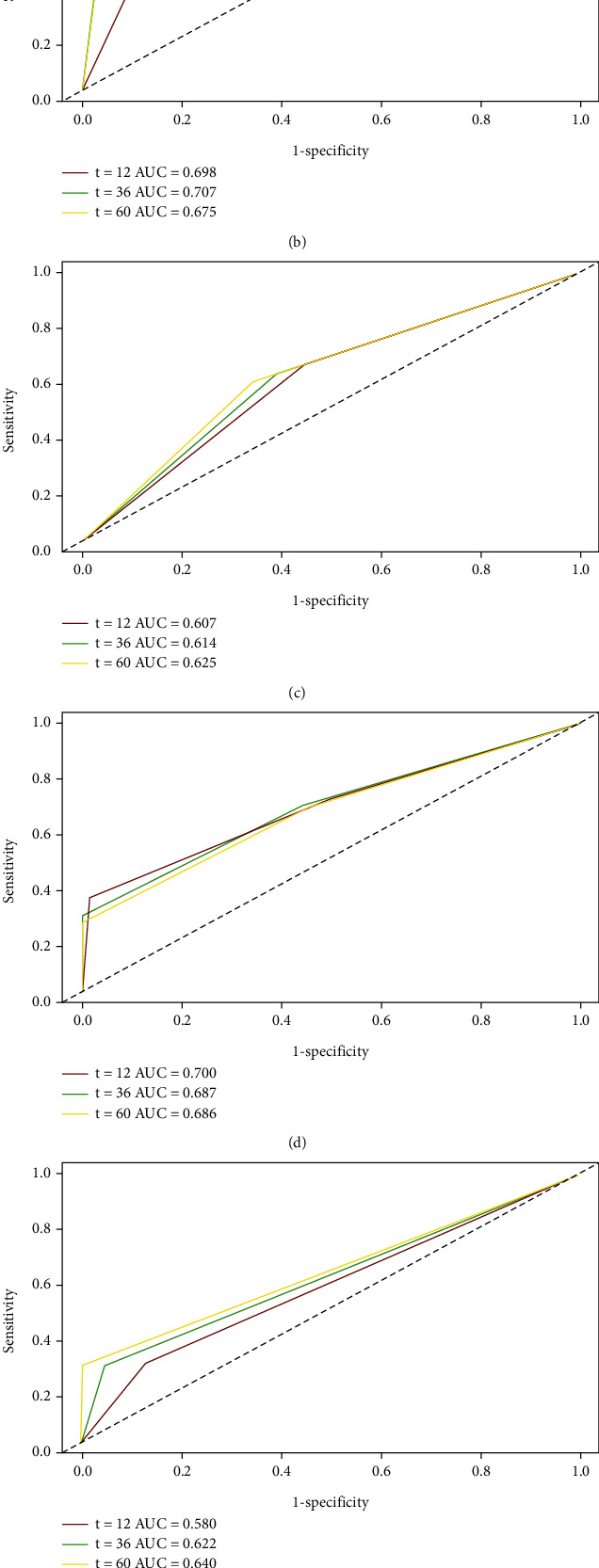
The time-dependent ROC of A-A score, ALP, and AFP on 1-, 3-, and 5-year OS and RFS. The AUC of A-A score on OS (a) and RFS (d) was higher than that of either ALP (b, e) or AFP (c, f). ROC: receiver operating characteristic.

Time-dependent receiver operating characteristic (ROC) curves can be used to compare the discrimination of the AFP and ALP scores in predicting prognosis [21]. The AUCs of A-A score to predict the 1-, 3-, and 5-year OS were 0.722, 0786, and 0.722 (AFP = 0.698, 0.707, and 0.625, respectively; ALP = 0.607, 0.614, and 0.625, respectively), and the AUCs of A-A score to predict the 1-, 3-, and 5 year RFS were 0.700, 0.687, and 0.686 (AFP = 0.580, 0.622, and 0.640, respectively; ALP = 0.565, 0.583, and 0.590, respectively) (Figure 5), suggesting that A-A score may be superior to AFP or ALP alone [22].

### 3.4. A-A Score Analysis

**Figure 6:**
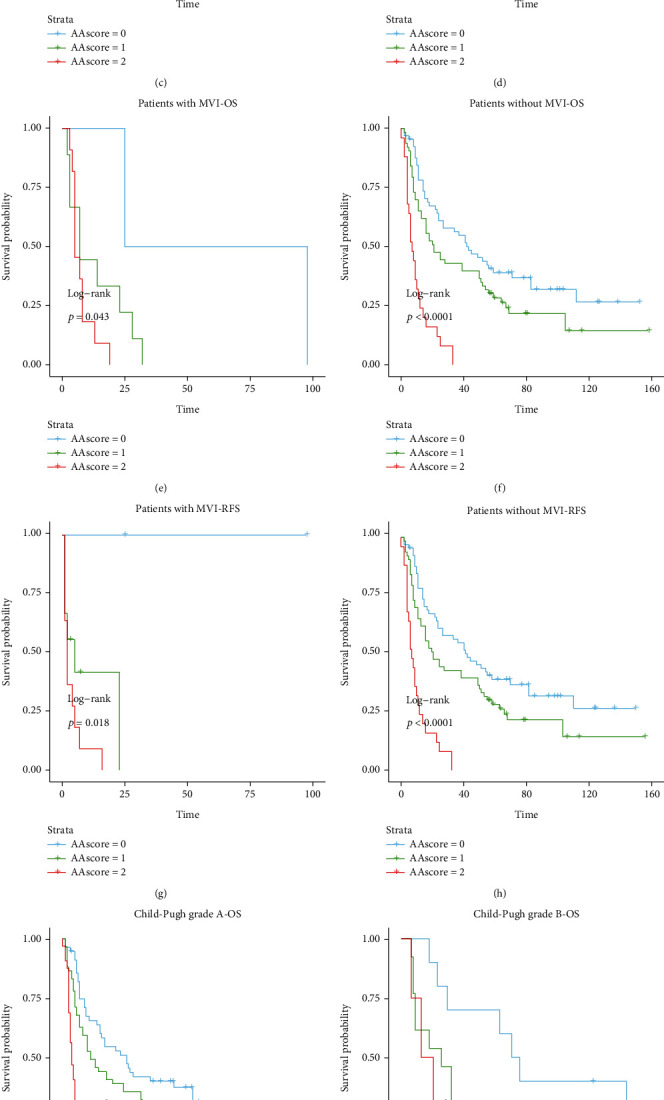
Subgroup survival analysis based on gender or MVI or child A and B. The A-A score could significantly stratify the prognosis of solitary HCC patients in different gender (a–d) patients with MVI (e–h) and patients with child A or B (i–l). MVI: microvascular invasion.

We performed the analysis according to the gender of postoperative patients with rHCC (Figures 6(a)–6(d)). We found that A-A scores had a good predictive ability in both genders. In particular, for male patients, the 1-, 3-, and 5 year OS rates were 78.0%, 55.9%, and 37.3% for patients with a score of 0; 62.3%, 41.0%, and 23.0% for patients with a score of 1; 24.0%, 0.0%, and 0.0% for patients with a score of 2 (*p* < 0.001). For female patients, the 1-, 3-, and 5-year OS rates were 75.0%, 50.0%, and 50.0% for patients with a score of 0; 63.6%, 18.2%, and 0.0% in patients with a score of 1; 33.3, 0.0, and 0.0% for patients with a score of 2 (*p* = 0.023). With statistical significance at 1, 3, and 5 years, the A-A score has the ability to predict the prognosis of patients of different genders.

In addition, having microvascular invasion (MVI) has been testified by some institutions as a poor prognostic factor for nrHCC and rHCC [22]. Patients who have MVI had a higher risk of postoperative recurrence and death. To further verify the predictive value of the A-A score, we analyzed the survivals based on the presence or absence of MVI according to the pathological reports of the patients. As shown in (Figures 6(e)–6(h)), our constructed score performed well in predicting the survival of patients with or without MVI. In particular, when patients had no MVI, the prognosis varied greatly between the different scores, with 1-, 3-, and 5 year RFS rates of 52.3%, 36.9%, and 23.1% in the score = 0 group and 52.4%, 30.2%, and 19.0% in the score = 1 group, respectively (*p* < 0.001).

We continued to grade the patients according to their Child-Pugh grade (Figures 6(i)–6(l)). We found that the A-A score prediction also performed well in patients with Child grades grade A and B rHCC (Child-Pugh grade C was excluded because there was only one patient in class C in the entire cohort). For patients with Child-Pugh grade A, the OS rates at 1, 3, and 5 years were 73.2%, 51.8%, and 35.7%, respectively, for patients with an A-A score of 1 and 62.7%, 39.0%, and 18.6%, for patients with an A-A score of 2.

## 4. Discussion

Tumor rupture is a severe complication of HCC, and it usually has a very poor prognosis. The probability of spontaneous rupture of HCC is 3% to 26% [5, 6, 13], and according to some studies, the mortality rate in patients who develop spontaneous rupture is as high as 32% to 6.7%. Some authors [13] have shown that the tumor length, the number of tumors, treatment before tumor rupture, alanine aminotransferase level, bicarbonate level, age, antitumor treatment during follow-up, and albumin level are prognostic factors for tumor rupture regardless of surgery. On the other hand, some investigators [11, 23] believe that patients with spontaneously ruptured HCC who undergo staged hepatectomy show similar long-term survival and recurrence patterns to patients with unruptured HCC who have similar tumor characteristics and liver function status, so surgery is recommended for rHCC. Li et al. [24] also believe that surgical resection should be the treatment modality for ruptured HCC patients in BCLC stages A and B. Since the proportion of ruptures is higher in the Asian region than in Europe and the United States, we believe that it is necessary to establish a simple scoring system to assess the postoperative prognosis of patients.

At present, many markers, such as AFP, CEA, and CA199, are used in the preoperative assessment [10, 25]. Some studies have shown that preoperative serum AFP or ALP plays a very important role in the diagnosis of rHCC and the prediction of postoperative prognosis, and higher preoperative serum AFP or ALP levels have been associated with poor postoperative prognoses. Different studies have different optimal cut-off values for preoperative serum AFP or ALP to predict the prognosis of HCC. For the selection of the AFP stage index, we directly selected the value from previous studies (that is, 1000 ng/mL). Because the selection of the cut-off value for ALP is controversial, in our study, the OS was dichotomized with preoperative serum ALP level as a continuous variable. We used X-tile software developed at Yale University and determined the optimal cut-off value for ALP as 92 U/L.

We found that the prognoses of patients with ALP > 92 U/L and <92 U/L were significantly different (Figure 4), and the same was true for AFP. Therefore, in this study, we combined AFP with ALP and used them to predict the prognosis of patients with rHCC. All ruptured HCC patients could be divided into three groups according to our A-A score. We set AFP > 1000 ng/mL as 1 point and AFP < 1000 ng/mL as 0 point and ALP > 92 U/L as 1 point and ALP < 92 U/L as 0 point. Patients with a total score of 2 accounted for 20.6%, those with a total score of 1 accounted for 41.1%, and those with a total score of 0 accounted for 38.3%.

Notably, patients with an A-A score of 2 had the worst outcome and those with an AA score of 0 had the best outcome in terms of the OS as well as the RFS. In addition, ROC analysis showed that A-A score had better discrimination than AFP or ALP alone as predictors, and it also had stronger clinical utility. Preoperative A-A score was an independent prognostic factor associated with OS and RFS in multivariate Cox regression models (HR: 2.28; 2.09). This could indicate that the A-A score has a definite role in predicting the postoperative prognosis of patients with tumor rupture. We can therefore define patients with a score of 0 as the low-risk group for recurrence and death after rHCC surgery, those with a score of 1, can be regarded as medium-risk, and those with a score of 2 can be regarded as high-risk.

We believe that this A-A score can be used by clinicians as an objective index to guide postoperative clinical decision-making in patients with rHCC. In particular, in terms of OS, when patients with rHCC were preoperatively assessed as having an AA score of 2, it can be seen from the survival curve that high-risk patients had an inferior prognosis after hepatectomy, with a median survival time of only six months. Therefore, we can screen such patients using an AA score before surgery. Using other treatment methods (such as TACE and RFA) may achieve a similar overall survival rate and reduce the pain caused by surgery. In particular, a meta-analysis showed [26] that the prognosis of HCC treated with TACE combined with RFA was equivalent to that treated with surgery. Alternatively, conservative treatment may also achieve a relatively similar prognosis for such patients.

Microvascular invasion (MVI) has been extensively studied as a poor prognostic factor for OS and RFS in nrHCC as well as in rHCC. [22, 27–30] Therefore, MVI has been considered a factor affecting postoperative rHCC in previous studies. At present, there has not been a study that grouped patients based on the presence of MVI. We divided postoperative patients into two groups based on whether they had MVI or not while using the A-A score to predict the postoperative prognosis in these two groups. Encouragingly, the A-A score showed good power in predicting the OS as well as the RFS in both groups.

Although the Child-Pugh grade was not statistically significant in multivariate regression, Child-Pugh grade is of great value in judging the prognosis of liver tumors. At the same time, we observed that the composition of the child grades was not the same in different scoring groups. Therefore, we also grouped the patients based on their Child-Pugh grade. Finally, we found that the A-A score can effectively predict the postoperative prognosis of patients with ruptured HCC in Child-Pugh grade A or Child-Pugh grade B. Child-Pugh grade is associated with the occurrence of postoperative complications. Among the patients we admitted, the proportion of males was still much more than that of females, about 6.94 : 1, and there was a significant difference in physiological status between males and females, so stratified analysis of gender showed that the predictive power of the A-A score in both sexes remained statistically significant, both OS and RFS.

Recently, pathological factors have been increasingly studied, including pathological differentiation type, which is also considered a factor affecting the OS and RFS in HCC patients. In univariate analysis, pathological differentiation type was statistically different. Unfortunately, this measure was not included in the multifactorial Cox model. Currently, more studies have used the Edmondson-Steiner grade as a grading guide, and from Table 1, we can know that the pathological grades of patients are also different at different scores. Pathological differentiation factors should therefore also be considered for the postoperative evaluation of patients with rHCC.

This research has some limitations. Firstly, this study was a retrospective single-center research. This scoring system should be externally validated to improve its applicability. Second, the sample size is still insufficient.

In conclusion, we established a scoring system (A-A score) based on the preoperative serum AFP and ALP levels. Our scoring system is simple and easy to use it only requires two variables, and it can achieve a very good discrimination effect and facilitate clinicians to make patient management decisions. We divided the A-A score into three groups, score 0 as the low-risk group, score 1 as the intermediate-risk group, and score 2 as the high-risk group, and there were significant differences in the survival curves among these three groups. We also performed discrimination tests and assessed the clinical utility of the A-A score by ROC found that this score had better discrimination compared to AFP and ALP alone. At the same time, we analyzed all patients according to three variables, and the A-A score also had good differentiation in different subgrades [27, 28].

## Figures and Tables

**Table 1:** Clinicopathological characteristics grouped by the A-A score.

Variables	Total	Group A (A-A score = 0)	Group B (A-A score = 1)	Group C (A-A score = 2)	*p*-value
	*n* = 175	*n* = 67	*n* = 72	*n* = 36	
Gender					0.464
Male	153 (87.4)	59 (88.1)	61 (84.7)	33 (91.7)	
Female	22 (12.6)	8 (11.9)	11 (15.3)	3 (8.3)	
Age	46 (38-54)	50 (41-54)	46 (37-54)	41 (32-53)	0.055
Tumor max length	7.4 (5.0-10.2)	5.9 (4.0-8.4)	7.6 (5.4-10.0)	10.1 (7.4-11.9)	<0.001
Tumor number					0.003
Single	136 (77.7)	60 (89.6)	54 (75.0)	22 (61.1)	
Multiple	39 (22.3)	7 (10.4)	18 (25.0)	14 (38.9)	
Portal hypertension					0.815
No	132 (75.4)	49 (73.1)	56 (77.8)	27 (75.0)	
Yes	43 (24.6)	18 (26.9)	16 (22.2)	9 (25.0)	
MVI					<0.001
No	153 (87.4)	65 (97.0)	63 (87.5)	25 (69.4)	
Yes	22 (12.6)	2 (3.0)	9 (12.5)	11 (30.6)	
Preoperative TAE					0.684
No	151 (86.3)	57 (85.1)	64 (88.9)	30 (83.3)	
Yes	24 (13.7)	10 (14.9)	8 (11.1)	6 (16.7)	
Regular excision					0.096
No	135 (77.1)	55 (82.1)	57 (79.2)	23 (63.9)	
Yes	40 (22.9)	12 (17.9)	15 (20.8)	13 (36.1)	
Extent of resection					0.239
R0	168 (96.0)	66 (98.5)	69 (95.8)	33 (91.7)	
R1	7 (4.0)	1 (1.5)	3 (4.2)	3 (8.3)	
BCLC stage					<0.001
A	106 (60.6)	52 (77.6)	45 (62.5)	9 (25.0)	
B	37 (21.1)	7 (10.4)	17 (23.6)	13 (36.1)	
C	28 (16.0)	6 (9.0)	9 (12.5)	13 (36.1)	
Edmondson-Steiner grade					0.001
I	20 (11.4)	16 (23.9)	3 (4.2)	1 (2.8)	
II	82 (46.9)	32 (47.8)	36 (50.0)	14 (38.9)	
III	40 (22.9)	12 (17.9)	17 (23.6)	11 (30.6)	
IV	33 (18.9)	7 (10.4)	16 (22.2)	10 (27.8)	
Child-Pugh grade					0.022
A	147 (84.0)	56 (83.6)	59 (81.9)	32 (88.9)	
B	28 (16.0)	10 (16.4)	13 (18.1)	4 (11.1)	
AFP					<0.001
≤1000 ng/mL	82 (46.9)	67 (100.0)	15 (20.8)	0 (0.0)	
>1000 ng/mL	93 (53.1)	0 (0.0)	57 (79.2)	36 (100.0)	
ALP					<0.001
≤92 U/L	124 (70.9)	67 (100.0)	57 (79.2)	0 (0.0)	
>92 U/L	51 (29.1)	0 (0.0)	15 (20.8)	36 (100.0)	
Tumor capsule					0.606
No	130 (74.3)	48 (71.6)	53 (73.6)	29 (80.6)	
Yes	45 (25.7)	19 (28.4)	19 (26.4)	7 (19.4)	
Satellite foci					0.582
No	86 (49.1)	35 (52.2)	32 (44.4)	19 (52.8)	
Yes	89 (50.9)	32 (47.8)	40 (55.6)	17 (47.2)	

Abbreviation: MVI: microvascular invasion; TAE: transcatheter arterial mbolization; BCLC: Barcelona Clinic Liver Cancer; AFP: alpha fetoprotein; ALP: alkaline phosphatase.

**Table 2:** Univariate and multivariate analyses of prognostic factors in OS after hepatectomy.

	Univariate analysis			Multivariate analysis		
	*p*	HR	95% confidence interval	*p*	HR	95% confidence interval
Gender Male/female	0.338	1.263	0.784-2.033			
Age Per 1 year	0.355	0.993	0.978-1.008			
Tumor max length Per 1 cm	<0.001	1.092	1.048-1.136			
Tumor number Multiple/single	<0.001	2.117	1.449-3.093			
Portal hypertension No/yes	0.072	1.413	0.970-2.059			
MVI No/yes	<0.001	2.662	1.673-4.237			
Preoperative TAE No/yes	0.989	1.003	0.625-1.611			
Regular excision No/yes	0.123	1.361	0.920-2.013			
Extent of resection R1/R0	<0.001	5.643	2.570-12.389	<0.001	4.219	1.889-9.421
BCLC stage C/B/A	<0.001	1.604	1.336-1.924	0.017	1.359	1.055-1.749
Child-Pugh A/B	0.861	0.965	0.643-1.446			
Edmondson-Steiner IV/III/II/I	0.001	1.322	1.124-1.555			
Tumor capsule No/yes	0.47	0.867	0.589-1.277			
Satellite foci No/yes	0.005	1.62	1.155-2.272			
A-A score 3/2/1	<0.001	2.28	1.773-2.933	<0.001	1.871	1.432-2.443

Abbreviation: MVI: microvascular invasion; TAE: transcatheter arterial mbolization; BCLC: Barcelona Clinic Liver Cancer.

**Table 3:** Univariate and multivariate analyses of prognostic factors in RFS after hepatectomy.

	Univariate analysis			Multivariate analysis		
	*p*	HR	95% confidence interval	*p*	HR	95% confidence interval
Gender Male/female	0.962	1.012	0.623-1.644			
Age Per 1 year	0.232	0.991	0.977-1.006			
Tumor max length Per 1 cm	<0.001	1.094	1.048-1.142	0.026	1.057	1.007-1.110
Tumor number Multiple/single	<0.001	2.092	1.420-3.081			
Portal hypertension No/yes	0.03	1.526	1.041-2.236	0.043	1.497	1.013-2.212
MVI No/yes	0.036	1.731	1.037-2.891			
Preoperative TAE No/yes	0.916	1.027	0.625-1.687			
Regular excision No/yes	0.111	1.372	0.930-2.023			
Extent of resection R1/R0	0.004	3.125	1.447-6.748			
BCLC stage C/B/A	<0.001	1.444	1.196-1.742			
Child-Pugh grade A/B	0.968	0.992	0.658-1.494			
Edmondson-Steiner IV/III/II/I	0.03	1.198	1.018-1.410			
Tumor capsule No/yes	0.078	0.691	0.458-1.043			
Satellite foci No/yes	0.067	1.373	0.978-1.923			
AA score 3/2/1	<0.001	2.09	1.615-2.705	<0.001	1.789	1.353-2.366

Abbreviation: MVI: microvascular invasion; TAE: transcatheter arterial mbolization; BCLC: Barcelona Clinic Liver Cancer.

## Data Availability

The datasets used and analyzed during the current study are available from the corresponding author on reasonable request.

## Abbreviations

AFP: Alpha-fetoprotein
ALP: Alkaline phosphatase
HCC: Hepatocellular carcinoma
rHCC: Ruptured hepatocellular carcinoma
ROC: Receiver operating characteristic curve
CT: Computed tomography
MRI: Magnetic resonance imaging
MDT: Multidisciplinary approach
RFA: Radiofrequency ablation
TAE: Transcatheter arterial embolization
TACE: Transcatheter arterial chemoembolization
TKI: Tyrosine kinase inhibitors
OS: Overall survival
RFS: Recurrence-free survival.
